# MOVEMENT ASSESSMENT BATTERY FOR CHILDREN-SECOND EDITION: THEORETICAL ADEQUACY OF A MOTOR ASSESSMENT INSTRUMENT

**DOI:** 10.1590/1984-0462/2022/40/2020205

**Published:** 2021-04-02

**Authors:** Patrik Felipe Nazario, Luciana Ferreira, Jorge Both, José Luiz Lopes Vieira

**Affiliations:** a Universidade Estadual de Maringá, Maringá, PR, Brazil.; b Universidade do Estado do Paraná, Paranavaí, PR, Brazil.; c Universidade Estadual de Londrina, Londrina, PR, Brazil.; d Universidad Católica del Maule, Talca, Chile.

**Keywords:** Motor activity, Educational measurement, Psychometrics, Child, Child, preschool, Atividade motora, Avaliação educacional, Psicometria, Criança, pré-escolar

## Abstract

**Objective:**

To investigate the adequacy of the theoretical model of the Movement Assessment Battery for Children-Second Edition (MABC-2) instrument.

**Methods:**

582 children, of both sexes, aged between 3 and 5 years and residents in the city of Maringá (state of Paraná, Southern Brazil) participated in the study. Data were collected from May/2014 to June/2015 and analyzed using descriptive and inferential statistics.

**Results:**

The evidence obtained from exploratory factor analysis indicated the presence of two factors, which was the option that best fitted the explanatory model. Hence, it was necessary to regroup the motor tasks of the dimensions “Aiming & catching” and “Balance” into only one dimension. It is noteworthy that the “Bicycle trail” motor task did not fit the model, as it presented a low and negative factor load in the analyzed dimensions. In the confirmatory factor analysis, adequate adjustment indices were observed for the tested model, which confirmed the non-classification of the “Bicycle trail” motor task in the original dimension.

**Conclusions:**

After removing the “Bicycle trail” motor task, the adjusted two-factor model seems to be the most appropriate to assess the motor performance of children participating in the study.

## RESUMO

**Objetivo:**

Investigar a adequabilidade do modelo teórico do instrumento de avaliação motora *Movement Assessment Battery for Children-Second Edition*.

**Métodos:**

Participaram do estudo 582 crianças, de ambos os sexos com idade entre 3 e 5 anos da cidade de Maringá, Paraná, no período de maio/2014 a junho/2015. Para analisar os dados, utilizou-se a estatística descritiva e inferencial.

**Resultados:**

As evidências obtidas por meio da análise fatorial exploratória indicaram a presença de dois fatores. Essa opção foi a que melhor se ajustou ao modelo explicativo. Com isso, foi necessário reagrupar as tarefas motoras das dimensões “lançar e receber” e “equilíbrio” em apenas uma dimensão. Destaca-se que a tarefa motora “caminho da bicicleta” não se adequou ao modelo, pois apresentou carga fatorial baixa e negativa nas dimensões analisadas. Na análise fatorial confirmatória, observaram-se índices de ajustamento adequados para o modelo testado, a qual confirmou o não enquadramento da tarefa motora “caminho da bicicleta” na dimensão original.

**Conclusões:**

Após a retirada da tarefa motora “caminho da bicicleta”, o modelo ajustado de dois fatores parece ser o mais adequado para avaliar o desempenho motor das crianças participantes do estudo.

## INTRODUCTION

The validity and reliability of the motor tests, used to discriminate the motor performance of typical and atypical children, are paramount regarding the quality of the instrument. Taking this into consideration, a measuring instrument must be valid and accurate.^1^ ^,^ ^2^

There is still no consensus on a “gold-standard” motor assessment. However, studies^3^ ^,^ ^4^ ^,^ ^5^ show that the *Movement Assessment Battery for Children-Second Edition* (MABC-2) is one of the tests used worldwide to identify children with developmental coordination disorder (DCD).^3^ ^,^ ^6^ ^,^ ^7^ This motor assessment instrument has been used in North America,^8^ ^,^ ^9^ Greece,^10^ ^,^ ^11^ Japan,^12^ Netherlands^13^, and Brazil.^5^ ^,^ ^14^ ^,^ ^15^

Although globally used, psychometric problems resulting from the use of MABC-2 were pointed out, for example, in Germany. Wagner, Kastner, Petermann and Bös,^16^ when analyzing the factorial validity of the MABC-2 test for children aged 7 to 10 years, found a problematic model in four motor tasks, confirming doubts about discriminant and convergent validities. In China,^17^ the authors concluded that the reproducibility and validity of MABC-2 age band 1 were poor, emphasizing the need to adjust part of the items to improve the psychometric properties of the test when applied to Chinese children aged 3 to 6 years. In Brazil,^18^ the analysis of the MABC-2 multidimensionality for children aged 7-10 years indicated that the exclusion of three subtests resulted in a better adjusted model. Therefore, there is a gap in the literature, mainly in Brazil, in terms of a detailed adequacy analysis of MABC-2 for children aged 3 to 5 years. This assumption was elucidated by Brown and Lalor,^19^ when they reviewed MABC-2 and argued that there are issues regarding the context, the translation of the items, and the assessment of one age group at a time.

Motor assessment studies using MABC-2 have a common feature: the use of standard scores, which arise from the validation process of the instrument based on a sample from the United Kingdom. It is noteworthy that, in the MABC-2 manual,^20^ there is no evidence related to the construct validity. The content validity was performed according to the assessment of an evaluation commission based on the motor tasks of the first version of MABC.^19^ Implications of the aforementioned aspects demonstrate the need for restructuring the test,^18^ removing some items from the test^17^, and the probable reduction in the frequency of error in the final classification of the DCD diagnosis in the evaluated children.^21^ Therefore, the present study investigated the adequacy of the theoretical model of the MABC-2 motor assessment instrument in Brazilian children aged 3 to 5 years.

## METHOD

The population available at the time of the study in 2014 was 6,278 children, aged between 3 and 5 years, enrolled in 54 municipal centers for early childhood education (*Centros Municipais de Educação Infantil* - CMEI). Then, the city of Maringá, in the state of Paraná, was divided into four regions (Northwest, Northeast, Southwest and Southeast), and a CMEI was drawn from each region, with the exception of the Northwest region, for which another CMEI was added for being the region with the largest number of children, totaling five CMEI. To obtain a representative sample, using Richardson’s formula^22^ and considering 5% of sampling error and 95% reliability, 362 children would be needed. The method for selecting children in each CMEI was simple random sampling, with the final sample consisting of 582 children (304 boys and 278 girls), aged between 36 and 71 months (mean=50.0, standard deviation [SD]=9.3).

MABC-2, by Henderson, Sugden and Barnett,^20^ was used to verify children’s motor performance. Motor tasks are grouped into the following categories: (1) manual dexterity, comprising the activities of posting coins, threading beads, and bicycle trail; (2) aiming and catching, involving activities of catching a beanbag and throwing a beanbag onto a goal; (3) balance, with the activities of one-leg balance, walking heels raised, and jumping on mats. For this study, the age band 1 of the instrument was considered. Raw data were measured on a time scale or number of errors/hits.

For data collection, the researchers, who were physical education professionals, were trained for one month, twice a week, in the domains of the different tasks of MABC-2. The intra- and inter-rater reliability was established for each task of the test by the intraclass correlation coefficient (ICC), with a 95% confidence interval (95%CI). Overall, the results showed very strong (ICC 0.91≤0.99; p<0.001) and strong (0.75≤0.90; p<0.001) correlations both intra- and inter-raters.

The research was approved by the Research Ethics Committee involving Human Beings of the university (Protocol No. 35712011), authorized by the Department of Education (SEDUC), with parents or guardians filling out the informed consent form. After drawing the schools, data collections were scheduled at the respective CMEI, with each child being individually assessed for 20 minutes.

The raw data were initially analyzed using descriptive statistics. To adjust the classification of motor performance in each motor task considering the logic that the higher the gross number, the lower the child’s motor performance, motor tasks were assessed by the number of hit/error and tabulated according to the number of errors. Thereafter, raw data were transformed into Z-scores, standardizing the measurement of motor tasks. To transform these Z-scores into a convenient analysis scale, the following formula was applied to convert the data into standard scores with a mean of 10 and SD of 3: new standard score=(Z-score)*(new SD )+new mean. This transformation follows the same procedures performed in the original standardization of the test, in which the scores range from 1 to 19.^20^ To verify the total performance of the children, the scores for each motor task of the new standardization were added, resulting in a scale with scores ranging from 67 to 139 and percentages from 0.05 to 99.50, respectively.

To avoid problems of sensitivity and normality of data in factor analysis, it was considered that items with asymmetry and kurtosis greater than 3 and 7, respectively, would be problematic for the analyses, as indicated by Marôco.^23^ However, all items met the aforementioned criteria. To identify the multivariate extreme cases, the Mahalanobis distance was used, excluding values above the adopted level of significance, considering the degrees of freedom of the model (df=9).

Reliability, the ability of an instrument to produce reliable results in different situations, was verified using Cronbach’s alpha and composite reliability, which must be higher than 0.7 to be considered acceptable.^23^ Furthermore, inter-item consistency was performed for similar motor tasks aimed at the same objective/dimension.^24^

To investigate the internal structure of the scale, exploratory factor analysis (EFA) was performed to reduce variables by factors with similar variances. The number of factors to be tested in the model was determined by eigenvalues (values higher than 1.00, according to the Kaiser criterion), analysis of the screen plot, commonalities, and factor interpretability (model with theoretical foundation). The unweighted least squares (ULS) method was employed with EFA, indicated for non-normal data, with the *Direct Oblimin* rotation, when there is correlation between factors, and the cutoff point of 0.40 was established for factor loads included in the model.^23^ The correlation matrix used in the model was obtained by Pearson’s correlation matrix.^23^

The models elucidated in EFA were tested in the confirmatory factor analysis (CFA). This procedure assesses the adjustment and adequacy of the model by quality indicators, factor loads, and individual item reliability. The estimation method employed was the ULS, due to the non-normality of the data. The used quality indicators of the model were: chi-square, Root Mean Square Error of Approximation [RMSEA] (values lower than 0.05 are considered adequate); Comparative Fit Index [CFI] (values higher than 0.95 are acceptable as good fitness); Goodness-of-fit Index [GFI] and Adjusted Goodness of Fit Index [AGFI] (values higher than 0.90 are acceptable); Tucker-Lewis Index [TLI] (acceptable when the value is higher than 0.97); Akaike Information Criteria [AIC], Bayesian Information Criteria [BIC] and Expected Cross-Validation Index [MECVI] (low values indicate a better model when compared with others).^23^ The average extracted variance for each construct was verified as suggested by Marôco.^23^

## RESULTS

The descriptive analysis of the results performed by mean, SD, percentage, and minimum and maximum values represents children who refused to perform any motor task (Table 1). It is noteworthy that children showed a greater refusal to perform the “bicycle trail” task (5.3%).

**Table 1:** Descriptive analysis of the raw scores of variables of the Movement Assessment Battery for Children-2.

	Dimension	n	Refusals (%)	Mean±SD	Minimum	Maximum
MD1a	Manual dexterity	576	1.0	14.0±6.0	7	80
MD1b		573	1.5	16.2±6.2	8	80
MD2		561	3.6	49.2±17.9	9	121
MD3		551	5.3	7.6±6.7	0	21
AC1	Aiming and catching	579	0.5	6.4±2.6	0	10
AC2		578	0.6	4.4±2.1	0	10
BL1a	Balance	573	1.5	9.3±8.4	0	30
BL1b		573	1.5	8.7±8.0	0	30
BL2		575	1.2	10.5±4.7	0	15
BL3		576	1.0	4.0±1.4	0	5

SD: standard deviation; a: preferred member; b: non-preferred member; MD1: posting coins (seconds); MD2: threading beads (seconds); MD3: bicycle trail (errors); AC1: catching a beanbag (hits); AC2: throwing a beanbag (hits); BL1: one-leg balance (seconds); BL2: walking heels raised (hits); BL3: jumping on mats (hits).

After verifying the number of refusals, it was decided to fragment the analysis in order to investigate the sex and age of children who refused to perform any motor task proposed by MABC-2 (Table 2). It is worth mentioning that 100% of the children who refused to perform the “bicycle trail” task (MD3) were 3 years old, and boys had the highest rates of refusals (68%). In general, 3-year-old children were the ones that most refused to perform the tasks, representing 86% of the refusals.

**Table 2:** Total number, per sex and age, of children who refused to do any motor task of the Movement Assessment Battery for Children-2 motor assessment instrument.

Instrument dimensions	Tasks	n total refusal	Sex		Age (years)		
			Boy n (%)	Girl n (%)	3 n (%)	4 n (%)	5 n (%)
Manual dexterity	MD1a	6	5 (83)	1 (17)	3 (50)	2 (33)	1 (17)
	MD1b	9	6 (67)	3 (33)	8 (89)	-	1 (11)
	MD2	21	14 (67)	7 (33)	16 (76)	5 (24)	-
	MD3	31	21 (68)	10 (32)	31 (100)	-	-
Aiming and catching	AC1	3	2 (67)	1 (33)	3 (100)	-	-
	AC2	4	2 (50)	2 (50)	4 (100)	-	-
Balance	BL1a	9*	7 (78)	2 (22)	9 (100)	-	-
	BL1b	9*	7 (78)	2 (22)	9 (100)	-	-
	BL2	7	6 (86)	1 (14)	7 (100)	-	-
	BL3	6	3 (50)	3 (50)	6 (100)	-	-
TOTAL		63	40 (63)	23 (37)	54 (86)	7 (11)	2 (3)

a: preferred member; b: non-preferred member; MD1: posting coins (seconds); MD2: threading beads (seconds); MD3: bicycle trail (errors); AC1: catching a beanbag (hits); AC2: throwing a beanbag (hits); BL1: one-leg balance (seconds); BL2: walking heels raised (hits); BL3: jumping on mats (hits); *same group of children.

After preliminary analyses, it was decided to remove all cases of refusal from the database, reducing the sample to 520 children. In addition, there were cases of outliers according to the Mahalanobis distance, with four more children being excluded from the database. Finally, 516 children were analyzed in the following steps. Table 3 shows mean, SD, and minimum and maximum values of the new standard scores, adjusted based on the selected sample, for the ten motor tasks.

**Table 3:** Mean, standard deviation, minimum and maximum values of the new standardized scores.

	Dimension	Mean±SD	Minimum	Maximum
MD1a	Manual dexterity	9.8±2.2	6	19
MD1b		9.9±2.6	2	19
MD2		10.0±2.9	3	19
MD3		9.9±2.9	6	18
Score of the manual dexterity dimension		29.8±5.9	17	48
AC1	Aiming and catching	10.0±2.9	2	13
AC2		10.0±2.9	3	17
Score of the aiming and catching dimension		20.0±4.9	6	31
BL1a	Balance	10.0±3.0	6	17
BL1b		10.0±3.0	6	17
BL2		10.0±2.9	2	12
BL3		10.0±2.9	1	12
Score of the balance dimension		30.0±6.3	12	42
Sum of scores of the dimensions		79.9±9.3	51	108

SD: standard deviation; a: preferred member; b: non-preferred member; MD1: posting coins (seconds); MD2: threading beads (seconds); MD3: bicycle trail (errors); AC1: catching a beanbag (hits); AC2: throwing a beanbag (hits); BL1: one-leg balance (seconds); BL2: walking heels raised (hits); BL3: jumping on mats (hits).

To verify the affinity of motor tasks between each other and with dimensions, the Spearman’s correlation analysis was performed. The results are demonstrated in Table 4.

**Table 4:** Correlation between tasks and dimensions of the Movement Assessment Battery for Children-2 motor assessment instrument.

	MD1a	MD1b	MD2	MD3	AC1	AC2	BL1a	BL1b	BL2	BL3	MD	AC	BL
MD1a	-										0.67*	-0.24*	0.16
MD1b	0.79*	-									0.63*	0.01	0.05
MD2	0.58*	0.54*	-								0.83*	-0.24*	-0.18*
MD3	-0.04	-0.08	0.20*	-							0.52*	-0.42*	-0.45*
AC1	-0.02	-0.01	-0.23*	-0.39*	-						-0.28*	0.80*	0.40*
AC2	-0.01	0.05	-0.15*	-0.31*	0.36*	-					-0.20*	0.82*	0.30*
BL1a	0.04	0.05	-0.15*	-0.47*	0.33*	0.28*	-				-0.25*	0.36*	0.67*
BL1b	-0.01	0.03	-0.26*	-0.49*	0.35*	0.25*	0.71*	-			-0.33*	0.35*	0.68*
BL2	-0.01	0.00	-0.15*	-0.28*	0.30*	0.23*	0.43*	0.46*	-		-0.20*	0.33*	0.79*
BL3	-0.05	-0.00	-0.09*	-0.23*	0.22*	0.19*	0.19*	0.19*	0.25*	-	-0.18*	0.24*	0.62*

**p<0.01; a: preferred member; b: non-preferred member; MD1: posting coins (seconds); MD2: threading beads (seconds); MD3: bicycle trail (errors); AC1: catching a beanbag (hits); AC2: throwing a beanbag (hits); BL1: one-leg balance (seconds); BL2: walking heels raised (hits); BL3: jumping on mats (hits); MD: manual dexterity dimension; AC: aiming and catching dimension; BL: balance dimension.

The tasks of the Aiming and Catching and Balance dimensions are interrelated. However, in the Manual Dexterity dimension, the “bicycle trail” task (MD3) has a low correlation with the “threading beads” task (MD2). When observing the correlations between motor tasks and dimension scores, it was found that the tasks of Aiming and Catching and Balance correlate with each other, but are negatively correlated with the Manual Dexterity dimension. These results indicate the possibility of having only two highlighted dimensions and, nevertheless, there is no correlation between the two dimensions.

**Figure 1:**
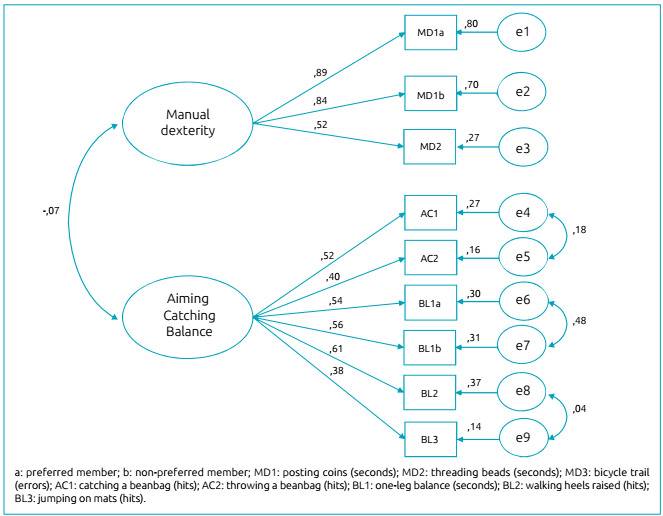
Two-factor model of the Movement Assessment Battery for Children-2 adjusted for a sample of 516 children.

As for the exploratory and confirmatory factor analyses of the MABC-2 instrument, the values of overall internal reliability and dimension-related reliability (Cronbach’s alpha) were initially verified based on the raw data. The results showed values lower than 0.7, indicating poor reliability of the instrument for the sample. The initial EFA indicated the existence of three possible factors. However, there was no consonance with the theoretical model originally proposed.^20^ It was verified that some tasks were allocated in dimensions whose names were not suitable to the content of the dimension proposed in the test. Furthermore, the “bicycle trail” task presented a negative factor load in all factors and was removed from the analysis, according to the evidence highlighted in the correlation analysis. Thus, EFA demonstrated two factors that could explain the variance of data, which were named as originally proposed. In fact, it was observed that items of the Aiming and Catching and Balance dimensions were grouped into a single factor. Table 5 shows the indices of the factor analysis with three and two factors (without the “bicycle trail” task) and the CFA. In Figure 1, the two-factor model of the MABC-2 adjusted for the sample is illustrated.

**Table 5:** Indices of exploratory and confirmatory factor analyses.

EFA							
Factor No.	KMO	Factor load	Communalities	Explained variance	RMSR	TLI	RMSEA
3	0.72	0.28-0.89	0.15-0.78	61%	0.03	0.92	0.07
2*	0.70	0.35-0.86	0.12-0.75	52%	0.06	0.84	0.10
CFA							
Factor No.	chi-square (df)	GFI/AGFI	RMSEA	TLI	CFI	RMR	Factor load
2*	80.32 (23)	0.96/0.93	0.07	0.92	0.95	0.56	0.38-0.89

EFA: Exploratory Factor Analysis; CFA: Confirmatory Factor Analysis; KMO: Kaiser-Meyer-Olkin; RMSR: Root Mean Square of the Residuals; TLI: Tucker Lewis Index of Factoring Reliability; RMSEA: Root Mean Square Error of Approximation; GFI/AGFI: Goodness of Fit Index/Adjusted Goodness-of-Fit Index; CFI: Comparative Fit Index; RMR: Root Mean Square; *without the “bicycle trail” motor task.

The standardized factor loadings of each item in the adjusted model were between 0.38 and 0.89 and statistically significant (p<0.001). It is noteworthy that the model was better adjusted after the correlation between the errors “e4” and “e5” (aiming and catching task), errors “e6” and “e7” (balance on dominant and non-dominant legs), and “e8” and “e9” (walking heels raised and jumping on mats). These correlations are theoretically justified by conceptually assessing the same motor skills. According to evidence elucidated based on the correlation matrix of the motor tasks, the most adequate theoretical model indicates the non-inter-construct relationship in MABC-2 for the sample. The convergent validity of the constructs was 0.75 (manual dexterity) and 0.50 (aiming, catching, and balance), which are values higher than those recommended in the literature.^21^

## DISCUSSION

The factor structure of the theoretical model of the motor assessment instrument (MABC-2) originally proposed by Henderson et al.^20^ did not fit the study sample. It became evident that the “bicycle trail” task did not fit the explanatory model, being excluded from the adjusted model. This motor task represented the greatest challenge for children due to the high number of refusals and the poor motor performance achieved (Table 2). Thus, it is reasonable to infer that such skill was negatively impacting the final result of the assessment, classifying children as having DCD, as the means of motor tasks are not weighted according to their degree of difficulty in the final classification. It should be noted that the authors found no study considering the question of weighting the MABC-2 motor tasks based on difficulty.

The elucidated evidences, concerning the low motor performance in the “bicycle trail” motor task, are similar to those found in studies conducted in Germany,^16^ Netherlands,^13^ and China,^17^ which highlighted that such task presented adequacy problems. In the study carried out in China, the authors removed the “bicycle trail” motor task from the set of skills, and the adjustment of the explanatory model was adequate, a fact similar to the present research. The study conducted in the Netherlands by Smits-Engelsman et al.^13^ showed that 3-year-old children had difficulties in performing the “bicycle trail” task, which presented the highest number of refusals in the sample of this study. These indications point to a possible inadequacy of this motor task for 3-year-old children.

A relevant question about studies whose authors investigated the factor model and conducted confirmatory analyses is that, usually, they use scores that were standardized based on the original sample of 1,172 children to perform the analyses. In Brazil, Valentini et al.^25^ stated the reliability and validity of the MABC-2 instrument for Brazilian children aged 3 to 13 years, although based on the standardized scores in the original sample.

In CFA, it was found that the “jumping on mats” task had a low factor loading in the dimension of aiming, catching, and balance. This evidence is in line with the study carried out in China on 1,823 children of the same age group.^17^ However, in the model adjusted to the sample, the factor load of the items in the respective constructs was considered moderate to high, suggesting that there is a substantial part of the variance of the dimensions that could be explained by the respective items. Indeed, the average extracted variance showed values greater than 0.50, which indicates adequate convergent validity.

The low reliability value (Cronbach’s alpha <0.7) has been reported in other studies,^13^ ^,^ ^26^ and the reliability value increased when the “bicycle trail” item was removed from the analysis, thus corroborating the evidence of the present study. Hua et al.^17^ stressed that the “bicycle trail” motor task presented a low correlation with the respective dimension, a fact evidenced in the present study. Such evidence supports the thesis of the need to first adapt motor tasks to the reality of each sample before classifying and diagnosing children’s motor performance. Another important result found in the EFA was the identification of items (“bicycle trail” and “jumping on mats”) whose commonalities and factor loads were below that recommended in the literature,^23^ indicating the small influence of the items on the final score of the instrument.

Creators of the MABC-2 instrument^20^ considered 15% of children with the worst motor performance to have a defined motor disorder or risk of DCD, i.e., children with scores ≤7, in the range of 1 to 19. From this perspective, the study conducted in the Netherlands^27^ standardized the motor task scores as the original study and the present study, on a scale of 1 to 19, with a mean of 10 and SD of 3. According to the results, if the cutoff point was maintained from the perspective of the original study, i.e., considering the standard score of 7 for diagnosing the disorder, there would be a greater number of children classified with DCD than expected, which could vary from 16.2 to 31.3% depending on the motor task assessed. However, in the original MABC-2 proposal, the percentage of children classified at risk or with DCD should be 15%, as intended by the authors and suggested by the American Psychiatric Association (APA),^28^ in which an index of 6% is evidenced as for schoolchildren with DCD worldwide. Thus, it is worth highlighting that, by maintaining the original standardization of the instrument, there is the possibility of underestimating the motor performance of the children in the present study, although a normative table for the children’s final classification has not been compiled.

While some authors consider MABC-2 to be a gold-standard motor assessment instrument^7^ ^,^ ^25^ to diagnose children with DCD, other authors emphasize the importance of finding more evidence, linked to technical adequacy, before using MABC-2 in isolation to identify children with DCD.^19^ ^,^ ^29^ Nevertheless, to date, there is no motor assessment instrument referenced by world-class criteria to diagnose children with DCD.^27^ Considering the literary controversy about the adequacy of the MABC-2 motor assessment instrument, in terms of the assessment of children’s motor performance aiming at diagnosing DCD, the instrument should be adequate for each study.

Some limitations of the present study must be considered for a better interpretation of the results. First, the sample was not stratified per sex and socioeconomic strata of children’s family. However, there was a sample representation for the city of Maringá (Paraná) and, as the participants were children aged from 3 to 5 years, the premise of equivalence of motor performance between sexes was assumed. Second, motor stimulation in the environments where children were inserted was not controlled. Finally, cultural differences were not assessed.

Further studies should be carried out in order to assess motor tasks according to the degree of difficulty and discriminatory power, in such a way to avoid motor tasks, such as “bicycle trail,” to have the same impact, for example, as the “jumping on mats” task, which accounted for a high proficiency index among the analyzed children. The results suggest that the MABC-2 instrument should be adjusted, i.e., the revision of the standardization of the items is necessary to improve the construct validity of the instrument. Moreover, the removal of the “bicycle trail” motor task may be associated with educational and cultural aspects and/or the lack of specific stimulus for this task. Adjusting the standardization of scores based on the sample seems to be adequate to better classify children, which can avoid false-positive DCD results. Studies with greater sample representativeness could consider the creation of standardization tables to better classify Brazilian children, based on the degree of difficulty and on the discrimination power of each motor task of MABC-2.

As practical applications for future studies, it is suggested the inclusion of a clinical sample of children who meet the four criteria for the diagnosis of DCD,^28^ aiming at the comparison with children who do not have a diagnosis of DCD, because the sensitivity and specificity of the instrument can be more reliably determined in a sample in which 50% of the included children have a diagnosis of DCD. In the meantime, only suggestions for preliminary practical applications can be made, for instance, the caution that clinical professionals must exercise when observing the final test result, mainly due to the lack of adjustment of the “bicycle trail” motor task to the model. For therapists and managers of intervention programs, there is the possibility of selecting specific content related to the low score obtained by children in one or more motor tasks of the MABC-2 instrument, and the longitudinal monitoring of children through periodic evaluations and reevaluations is recommended.
